# Supplementary material for: Glucose and Fructose Supplementation and Their Acute Effects on Anaerobic Endurance and Resistance Exercise Performance in Healthy Individuals: A Double-Blind Randomized Placebo-Controlled Crossover Trial
Source: Nutrients. 2022 Dec 2;14(23):5128. doi: 10.3390/nu14235128 (PMC9736485; doi:10.3390/nu14235128)
Supplement: Supplementary file 1 [file nutrients-14-05128-s001.zip › nutrients-2046906-supplementary.pdf]

Supplemental Material S1:

Participant inclusion criteria:

Inclusion Criteria:

- Informed consent obtained
- Male or female aged 18–65 years (both inclusive)
- Body mass index 18.0–29.9 kg/m^2 (both inclusive)
- Body Mass specific oxygen uptake >20 $ml/min/Kg^{-1}$ (VO_{2max})
- Sufficient lower- and upper body strength to conduct movements in leg press and chest press exercise (100% BM leg-press and 30%/75% BM chest-press)
- Normal glucose tolerance (measured via overnight fasting blood glucose levels)

Exclusion Criteria:

- Enrolment in other study
- Known or suspected hypersensitivity to trial product(s) or related products
- Receipt of any investigational medicinal product within 1 week prior to screening in this trial
- Suffer from or history of a life-threatening disease (i.e. cancer judged not to be in full remission except basal cell skin cancer or squamous cell skin cancer), or clinically severe diseases that directly influence the study results, as judged by the Investigator. This does not prohibit the participation of patients taking medications that influences the metabolism (e.g. statin) or cardio-respiratory system (e.g. asthma spray) as long as the therapy is stable and is not adapted throughout the run of the trial. Furthermore, it does not excluded patients how have celiac disease (or similar diseases or allergies), as long as the disease is stable, and patients are able to stay on their specific (e.g.) gluten-free diet.
- Participant with a heart rate <35 beats per minute (bpm) at screening (after resting for 5 min in supine position)
- Supine blood pressure at screening (after resting for 5 min in supine position) outside the range of 90–150 mmHg for systolic or 50–95 mmHg for diastolic (excluding white-coat hypertension; therefore, if a repeated measurement on a second screening Visit shows values within the range, the participant can be included in the trial). This exclusion criterion also pertains to participants being on anti-hypertensives
- Significant abnormal ECG at screening, as judged by the Investigator
- Any chronic (metabolic) disorder or severe disease which, in the opinion of the Investigator might jeopardise participant's safety or compliance with the protocol
- History of multiple and/or severe allergies to drugs or foods or a history of severe anaphylactic reaction

- Participant with mental incapacity or language barriers precluding adequate understanding or cooperation or who, in the opinion of their general practitioner or the Investigator, should not participate in the trial
- Any condition that would interfere with trial participation or evaluation of results, as judged by the Investigator
- Female of childbearing potential who is pregnant, breast-feeding or intend to become pregnant or is not using adequate contraceptive methods (adequate contraceptive measures include sterilisation, hormonal intrauterine devices, oral contraceptives, sexual abstinence or vasectomised partner).
